# Robotic Radiosurgery for the Treatment of Intramedullary Spinal Cord Metastases: A Case Report and Literature Review

**DOI:** 10.7759/cureus.609

**Published:** 2016-05-13

**Authors:** Rafael Garcia, Kita Sallabanda, Iciar Santa-Olalla, Jose Luis Lopez Guerra, Lijia Avilés, Morena Sallabanda, Eleonor Rivin, José Samblás

**Affiliations:** 1 Robotic Radiosurgery Unit, Imoncology; 2 Radiosurgery, Imoncology; 3 Radiation Physics, Imoncology; 4 Radiation Oncology, Virgen del Rocío University Hospital; 5 Master in Advanced Technological Applications in Radiation Oncology, University of Murcia and Imoncology Foundation; 6 Radiation Oncology, Hospital Universitario Puerta de Hierro; 7 Radiation Oncology, Institut de cancérologie Gustave Roussy

**Keywords:** robotic radiosurgery, intramedullary spinal cord metastases, radiosurgery

## Abstract

Modern technologies allow the delivery of high radiation doses to intramedullary spinal cord metastases while lowering the dose to the neighboring organs at risk. Whether this dosimetric advantage translates into clinical benefit is not well known. This study evaluates the acute and late toxicity outcomes in a patient treated with robotic radiosurgery for an intramedullary spinal cord metastasis.

A 50-year-old woman diagnosed in May 2006 with invasive ductal carcinoma of the right breast T2N3M1 (two liver metastases) received chemotherapy with a complete response. Subsequently, she underwent adjuvant whole-breast radiotherapy, along with tamoxifen. After several distant relapses, treated mainly with systemic therapy, the patient developed an intramedullary lesion at the C3-C4 level and was referred to our CyberKnife unit for assessment. A total dose of 14 Gy prescribed to the 74% isodose line was administered to the intramedullary lesion in one fraction. One hundred and two treatment beams were used covering 95.63% of the target volume. The mean dose was 15.93 Gy and the maximum dose, 18.92 Gy. Maximum dose to the spinal cord was 13.96 Gy, V_12_ ~ 0.13 cc and V_8_ ~ 0.43 cc.

Three months after treatment, magnetic resonance imaging showed a reduction in size and enhancement of the intramedullary lesion with no associated toxicity. During this period, the patient showed a good performance status without neurological deficits. Currently, with a follow-up of 37 months, the patient has the ability to perform activities of daily life.

Intramedullary spinal cord metastases is a rare and aggressive disease, often treatment-refractory. Our case demonstrates that radiation therapy delivery with robotic radiosurgery allows the achievement of a high local control without adding toxicity.

## Introduction

The spine is a common location for bone metastases. Approximately one-third of cancer patients develops bone metastases during the course of their disease. However, intramedullary metastases (IM) are rare, with a prevalence between 0.9 – 2.1% [1]. The cervical spine is the most common location for IM. The primary tumor is most commonly located in the lung, followed by breast cancer, melanoma, and renal carcinoma [1]. The presence of IM is usually the result of a fast, progressive, and systemic spread of the disease, predicting a poor prognosis and a short survival. Therefore, studies typically focus on data concerning short-term pain relief, which explains the fact that conventional radiation therapy at low doses and the use of corticosteroids have been the norm for inoperable patients until now.

In a recent review of 36 publications, 85 patients with IM from breast cancer were described [2]. The breast represented the second highest source of IM after lung cancer (26.5% versus 45%). Surgery was the primary treatment, and patients with metastasis from breast cancer had higher survival than patients with pulmonary cancer metastases (P = 0.05).

Stereotactic body radiotherapy (SBRT) has gained acceptance as a treatment modality for tumors of the spinal cord. Its role in the management of oligometastases, radioresistant metastases, and/or recurrent tumors is on the rise. However, the biggest experience with SBRT is described in bone lesions, with several retrospective studies and a small number of prospective studies that have reported excellent clinical results and low toxicity. The effectiveness of radiosurgery for IM has been reported in the case of hemangioblastomas, arteriovenous malformations, and ependymomas; however, the literature concerning intramedullary lesions of metastatic origin is still limited [3].

The CyberKnife® robotic radiosurgery system (Accuray, Inc., Sunnyvale, CA) is a non-invasive alternative to surgery for the treatment of tumors anywhere in the body, including IM. The treatment delivers beams of high-dose radiation to tumors with extreme accuracy. Whether this dosimetric advantage translates into clinical benefit is not well known. Thus, we review our experience treating an intramedullary metastasis with radiosurgery to assess its safety and efficacy and to define preliminary treatment recommendations. Informed patient consent was obtained for her treatment.

## Case presentation

A 50-year-old woman was diagnosed in May 2006 with an invasive ductal carcinoma in the right breast pT2N3, according to the clinical Tumor, Node, Metastasis (TNM) staging system (American Joint Committee on Cancer Staging Manual, 6th edition, 2002), subtype luminal A, estrogen receptors 20% and progesterone receptors 40%. The HER2/neu was negative, Ki-67 was 10%, and p53 was negative. Two lesions, compatible with liver metastases, were observed in the staging magnetic resonance imaging (MRI) and positron emission tomography–computed tomography (PET-CT). The patient received systemic therapy (Taxotere, adriamycin, and cyclophosphamide), achieving a complete response. Subsequently, the patient underwent adjuvant whole-breast radiotherapy encompassing the lymph nodes, along with a course of tamoxifen.

At her one-year follow-up, progression to the liver was observed, and determination of HER2 fluorescence in situ hybridization levels was repeated, with a positive result in patched form. Therefore, systemic treatment with vinorelbine, gemcitabine, and trastuzumab was prescribed. After six months, the patient developed bone progression (T12, L3, and left iliac blade). The patient received a cycle of Herceptin, lapatinib, and capecitabine chemotherapy, and a PET-CT reevaluation showed a partial response. At the 18 month follow-up, a radiological evaluation showed a complete response, and maintenance chemotherapy was prescribed with good tolerance. Following a disease-free survival of nine months, a PET-CT showed a lesion at the edge of the left iliac wing with a standardized uptake value of 5.4, as well as hypodense activity without right parietal activity.

In April 2010, a follow-up MRI showed a right frontoparietal multicystic 3 cm lesion, a left lateral caudate 12 mm lesion, and a retrocerebellar extra-axial mass of 2 mm, suggestive of a dural metastasis, as well as another cerebellar metastasis. After surgical resection of both the parietal and cerebellar lesions, histology of a triple positive breast adenocarcinoma with a high rate of proliferation was confirmed. The patient received whole brain radiotherapy with preservation of the scalp using helical tomotherapy (HT) and subsequent radiosurgery to the cerebellar lesion. During this time, the patient continued with trastuzumab, exemestane, and lapatinib for five months at which point bevacizumab was then added.

In January 2011, an increased uptake and an enlargement of the left iliac blade lesion were observed in a PET-CT. This lesion received treatment with HT, continuing with the same scheme of chemotherapy. At the six-month follow-up, there was an increased tumor marker level with normal imaging tests. Brain and cervical spinal cord MRIs were performed in January 2012, observing two millimetric lesions in the right cerebellar hemisphere and left anterior basal ganglia, along with an intramedullary lesion at the C3-C4 level. Exemestane was suspended and vinorelbine and an anti-HER agent were added. In March 2012, the patient was referred to our CyberKnife radiosurgery unit for assessment of treatment to the new lesions.

### Treatment description

**Figure 1:**
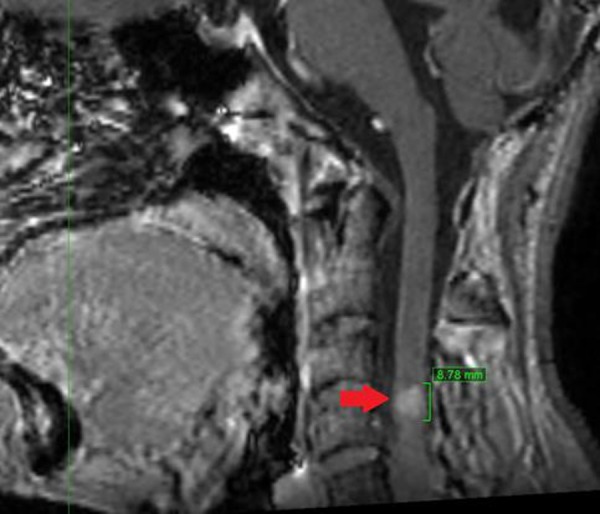
Sagittal view of magnetic resonance imaging, slice thickness 1.2 mm, lesion at C3-C4 level marked with red arrow

Immobilization of the patient's head in a supine position was performed using a thermoplastic mask. A craniocervical CT with and without contrast, slices of 1.25 mm thickness, along with brain and cervical MRI scans with gadolinium with 1.2 mm slices, were acquired for planning purposes. In these imaging studies, after consulting with a team of radiologists, only an intramedullary lesion at C3-C4 was observed without detection of the previously noted lesion in the left region of the basal ganglia. There was also a reduction in the size of the cerebellar hemisphere mass with lower uptake as compared with the MRI of January 2012. Due to these new findings and with the patient’s consent, it was decided to treat only the intramedullary lesion (Figure 1).

Our goal with this treatment was to eliminate the intramedullary lesion without an associated radio-induced myelopathy. In order to achieve this, the patient was treated with the CyberKnife® robotic radiosurgery system.

The organ at risk was a volume of spinal cord defined as 5.6 mm above and below the upper and lower limit of the lesion. Gross tumor volume (GTV) was defined as the macroscopic lesion visible on the MRI (0.167 cc) and the planning target volume (PTV) was equal to the GTV.

**Figure 2:**
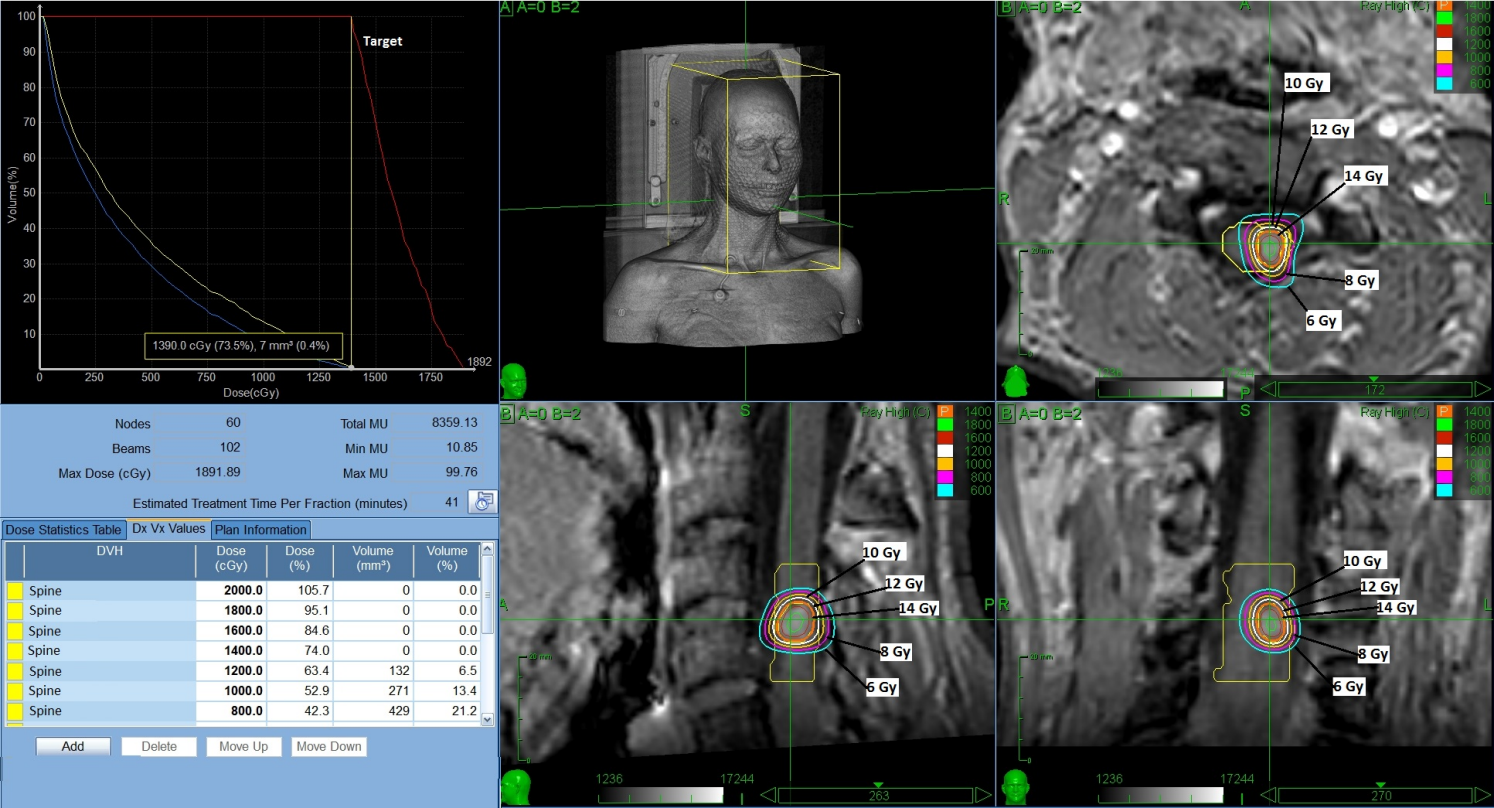
Dose-volume histogram and dose distribution

The treatment plan was generated with the Multiplan® inverse planning software, version 4.5.0, and delivered using the Xsight-Spine® Tracking System (Accuray, Inc., Sunnyvale, CA), which allows for correction, in real-time, of spine and tumor motion. A single dose of 14 Gy prescribed to the 74% isodose line was administered to the intramedullary lesion. This dose was chosen in order to decrease the risk of severe toxicity within this location. One hundred and two treatment beams were used, covering 95.63% of the target volume. The mean dose was 15.93 Gy and the maximum dose was 18.92 Gy. Maximum dose to the spinal cord was 13.96 Gy, V_12_ ~ 0.13 cc and V_8_ ~ 0.43 cc. The conformity index (defined as the ratio of total tissue volume that receives the prescription isodose or more to tumor volume that receives the prescription isodose or more) was 1.0 and homogeneity index (defined as the ratio of the maximum dose to the prescription dose) was 1.35 (Figure 2).

The approximate treatment time was 35 minutes. The treatment was completed without incident and with good tolerance. Prophylactic dexamethasone was prescribed with a tapering dosage.

### Follow-up

Three months after treatment, a reassessment brain-cervical MRI showed a reduction in size and enhancement of the intramedullary lesion with no associated toxicity. In addition, an increase in size and uptake of the left frontal horn lesion, compatible with a secondary lesion, was detected. The patient received treatment with the CyberKnife® to that intracranial lesion using 18 Gy to the 90% isodose line in a single fraction.

**Figure 3:**
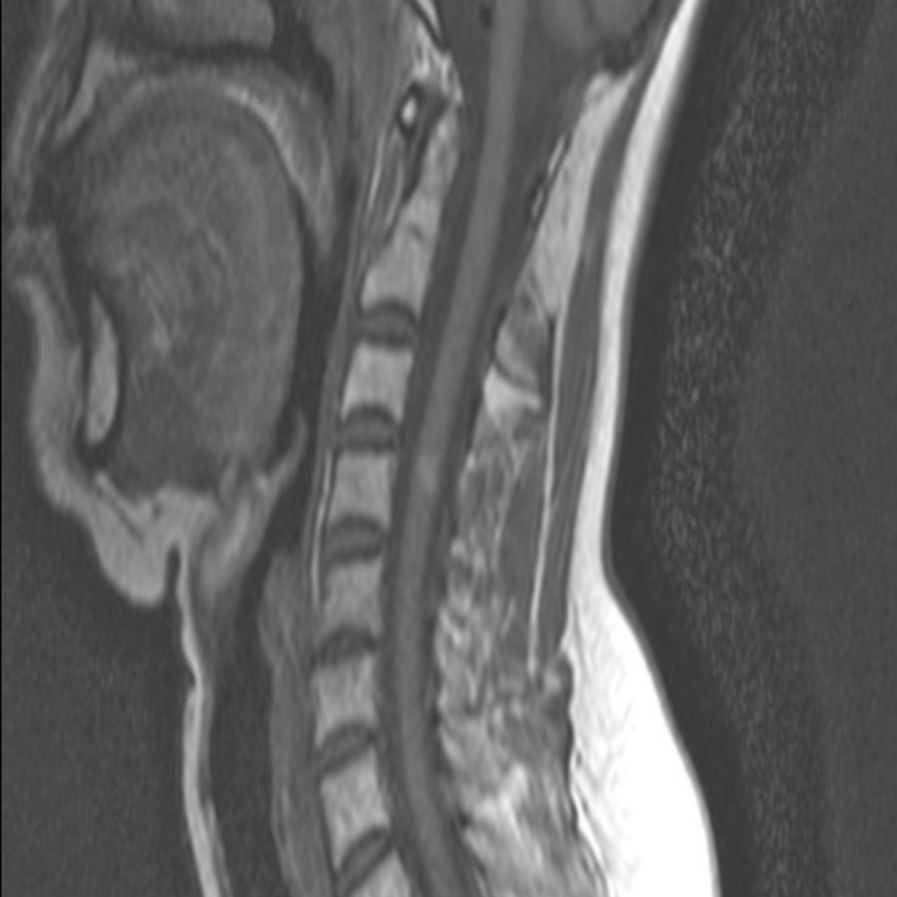
Magnetic resonance imaging 21 months after stereotactic radiosurgery

During this period, the patient showed a good clinical outcome without neurological deficits. In the follow-up MRI scan of December 2013, no tumor growth and no symptomatic myelopathy were observed (Figure 3).

**Figure 4:**
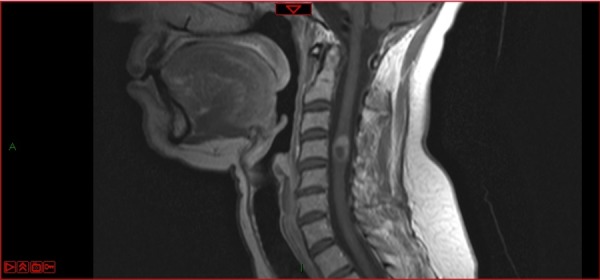
Radiation necrosis in the target lesion 31 months after robotic radiosurgery

Although the MRI of October 2014 showed radiological changes (radiation necrosis) in the tumor image, there was no associated symptomatology (Figure 4).

Currently, with a follow-up of 37 months, the patient has the ability to carry out the activities of daily living.

## Discussion

Intramedullary metastases are usually the result of rapid and progressive systemic disease. Breast cancer accounts for one of the solid tumors most commonly associated with the development of IM, after lung cancer. Technological developments in neuroimaging will lead us to a higher rate of detection of these types of metastases. The treatment of IM is complex and often depends on the location of the metastasis, systemic disease, life expectancy, and functional capacity. In the cases of IM after breast cancer, the treatment options are open surgery, chemotherapy, hormonal therapy, and radiotherapy.

A literature review of more than 200 stereotactic body radiation therapy spine articles from the past 20 years found only a single article that provided dose-volume data and outcomes for each spinal cord of a clinical dataset [4]. That article contains the first 102 stereotactic body radiation therapy spine treatments using the CyberKnife® in 74 patients from Stanford University Medical Center [5]. In all, 50 of the patients were previously irradiated to a median dose of 40 Gy in 2-3 Gy fractions and three patients developed treatment-related myelopathy. These dose-volume data were digitized into the dose-volume histogram Evaluator software tool where parameters of the probit dose-response model were fitted using the maximum likelihood approach [4]. Based on this limited data set, the unified low-risk dose tolerance limits for de novo cases yielded an estimated risk of spinal cord injury of ≤ 1% in 1-5 fractions, and the high-risk limits yielded an estimated risk of ≤ 3%. The Quantitative Analysis of Normal Tissue Effects in the Clinic (QUANTEC) Dmax limits of 13 Gy in a single fraction and 20 Gy in three fractions had less than 1% risk estimated from this dataset. In the previously irradiated cohort, the estimated risk levels for 10 and 14 Gy maximum cord dose limits in five fractions were 0.4% and 0.6%, respectively.

While hypofractionation can be performed with conventional radiotherapy devices, its limitations result from not allowing the delivery of ablative doses per fraction and not sparing the healthy intramedullary tissue from the radiation beam, which is an essential requirement in dose escalation. Safe dose escalation constitutes an increasingly important objective in oligometastatic patient treatment since many of these patients will live beyond traditionally described time-frames due to the availability of new and more effective systemic agents.

Gasser, et al. published their experience on surgical treatment in IM [6]. Thirteen patients were identified (seven adenocarcinomas, three poorly differentiated carcinomas, and three sarcomas). In those patients who underwent standard microsurgery, the average time to progression was 13 weeks and average survival was 31 weeks. All poorly differentiated tumors and sarcomas were resected incompletely and the surgical radicalism arose as a negative predictive factor for functional outcome, meaning that greater radicalism involved greater functional impairment.

The review of IM from breast cancer by Rostami, et al. concerning 85 patients presented from 36 publications found that survival is higher in patients who had debulking surgery compared with those who had no surgical treatment (6.3 vs. 4.1 months) [2]. However, only two of the 36 reviewed publications were radiosurgical series.

As we have seen so far, the treatment options were limited to palliative radiotherapy or effective surgery only for selected patients. Therefore, SBRT and stereotactic radiosurgery (SRS) emerge as interesting treatment modalities for patients with spinal metastases. The aim of spinal SBRT is to deliver high doses of radiation that could be considered locally therapeutic in single or up to five fractions, directed to the involved vertebrae or, in IM cases, to the portion of compromised spinal cord.

The existing data concerning SBRT for spinal lesions mainly comes from the experience of retrospective series on lesions that affected the vertebral body. A Phase I/II prospective study reported 74 spinal lesions treated with SBRT demonstrated that spinal toxicity could be avoided when the maximum dose to the cord was restricted to less than 10 Gy [7]. Unfortunately, there are very few references regarding the treatment of IM, probably because their incidence is uncommon [1]. Nevertheless, modern imaging studies allow us to improve the detection of these types of lesions.

The greater amount of information about the tolerable dose in the spine when using radiosurgery to treat intramedullary lesions comes from experience in treating benign disease. In fact, the series of Daly, et al. represents a study that reported the highest dose-tolerance of the spinal cord in humans [3]. Nineteen patients with hemangioblastomas were treated with a mean dose of 20 Gy (range: 18 – 30 Gy) and 10 lesions were treated using 18 to 25 Gy in two to three fractions. The mean maximum dose in a single fraction was 22.7 Gy (range: 17.8 – 39.9 Gy), average V_10_ was 0.454 cc (range: 0.226 – 3.543 cc), and the average dose to 0.500 cc of the spinal cord was 9.5 Gy (range: 5.3 – 22.5 Gy). Toxicities observed included a case of Grade 2 unilateral foot drop syndrome after five months and two cases of Grade 1 sensory deficit after two months. The actuarial estimated local control in three years was 86%. The conclusion was that, despite having exceeded the commonly cited constraints to the cord, the delivery of high doses to a small volume of the spinal cord can be done safely and effectively.

In terms of experience with metastatic lesions, only case reports involving a single patient and case series are available. Shin, et al. retrospectively reviewed nine patients with 11 IM treated with SRS at the Henry Ford Hospital [8]. The average age at presentation was 50 years, with a range of 14 to 71 years. There were four extramedullary-intradural lesions and seven intramedullary lesions. The mean treatment dose was 13.8 Gy (range: 10 - 16 Gy). The median duration of follow-up was 10 months. The symptoms improved in eight (80%) of the 10 evaluable lesions, one case remained unchanged, and one worsened in one instance. The median overall survival was eight months and no clinically significant toxicity was detected during the follow-up period.

The review of Veeravagu, et al. is the latest and largest series of patients with IM reported. The outcome of 11 lesions in nine patients treated with the CyberKnife® at Stanford Hospital was evaluated [9]. The sizes of the tumors were 0.12 − 6.4 cc (average: 4.8 cc), and all patients had neurologic deficits and other multiple metastases. The delivered doses were 14 to 27 Gy in one to five fractions with a biologically equivalent dose from 38 to 45 Gy. The largest volume was 6.4 cc, treated less aggressively to an approximate 22 Gy biologically effective dose (BED). One of the patients survived 14 months, but patients in the rest of the series had a mean survival of four months. The poor survival was attributed to systemic disease.

The key to SBRT in the spinal cord is to determine the position of the target while the patient is immobilized before the delivery of treatment and to ensure that it is in the same position at the time of the treatment delivery. Therefore, an image-guided system, which may include CT or stereoscopic x-ray systems, is required. The strategy of how to achieve this target, the dosimetry, and the contouring of the spinal cord has been recently reviewed. Ryu, et al. defined the local volume of the spinal cord, for calculation purposes, as 6 mm above and below the upper and lower limits of the white volume and concluded that intramedullary radiosurgery should limit the dose to 10 Gy to a 10% local spinal cord bounded volume [10]. On the other hand, others have proposed that the exposed volume should be limited to no more than 1 cc or an equivalent dose of 8 Gy in the spinal cord.

In the CyberKnife® system, a series of x-rays are used during treatment to detect the movements of the spine by its tracking method (X-Sight® Spine) that allows adjusting of the beam in real time. Another advantage is the high conformality of the dose that is reached in the vicinity of the target, keeping the exposed medullary tissue surrounding the lesion to a minimum.

## Conclusions

Spinal SBRT or SRS requires accuracy in both treatment planning and delivery. High-quality magnetic resonance (MR) support is very important in order to accomplish this. CT-MRI fusion improves treatment planning because the CT scan alone is considered suboptimal for the visualization of intramedullary lesions.

Although the available literature concerning the treatment of intramedullary lesions with radiosurgery is still limited, published experience so far presents SBRT as a safe and effective option in the local control of intramedullary lesions. In our experience, the treated patient achieved a complete response without neurological deficits secondary to radiosurgery.

We strongly believe that SBRT or SRS can be used in the treatment of IM without myelopathy and with good control of the pathology. This treatment has become the treatment of choice for the intramedullary metastases in our unit.
